# Supplementary material for: Predicting visual function by interpreting a neuronal wiring diagram
Source: Nature. 2024 Oct 2;634(8032):113–23. doi: 10.1038/s41586-024-07953-5 (PMC11446822; doi:10.1038/s41586-024-07953-5)
Supplement: Supplementary file 6 — CRF and ERF predictions for individual TmY4 and TmY9 cells. Analogous to Supplementary Data 3, but for TmY target types. Shown are the top four monosynaptic pathways, the strongest pathway passing through each of the top ten intermediary types (ranking from Extended Data Fig. 7), and the trisynaptic pathway Tm1–TmY–Dm3–TmY (see the section entitled Prediction of spatial normalization). [file 41586_2024_7953_MOESM6_ESM.zip › DataS4/TmY9qΓèÑ/720575940611830451.pdf]

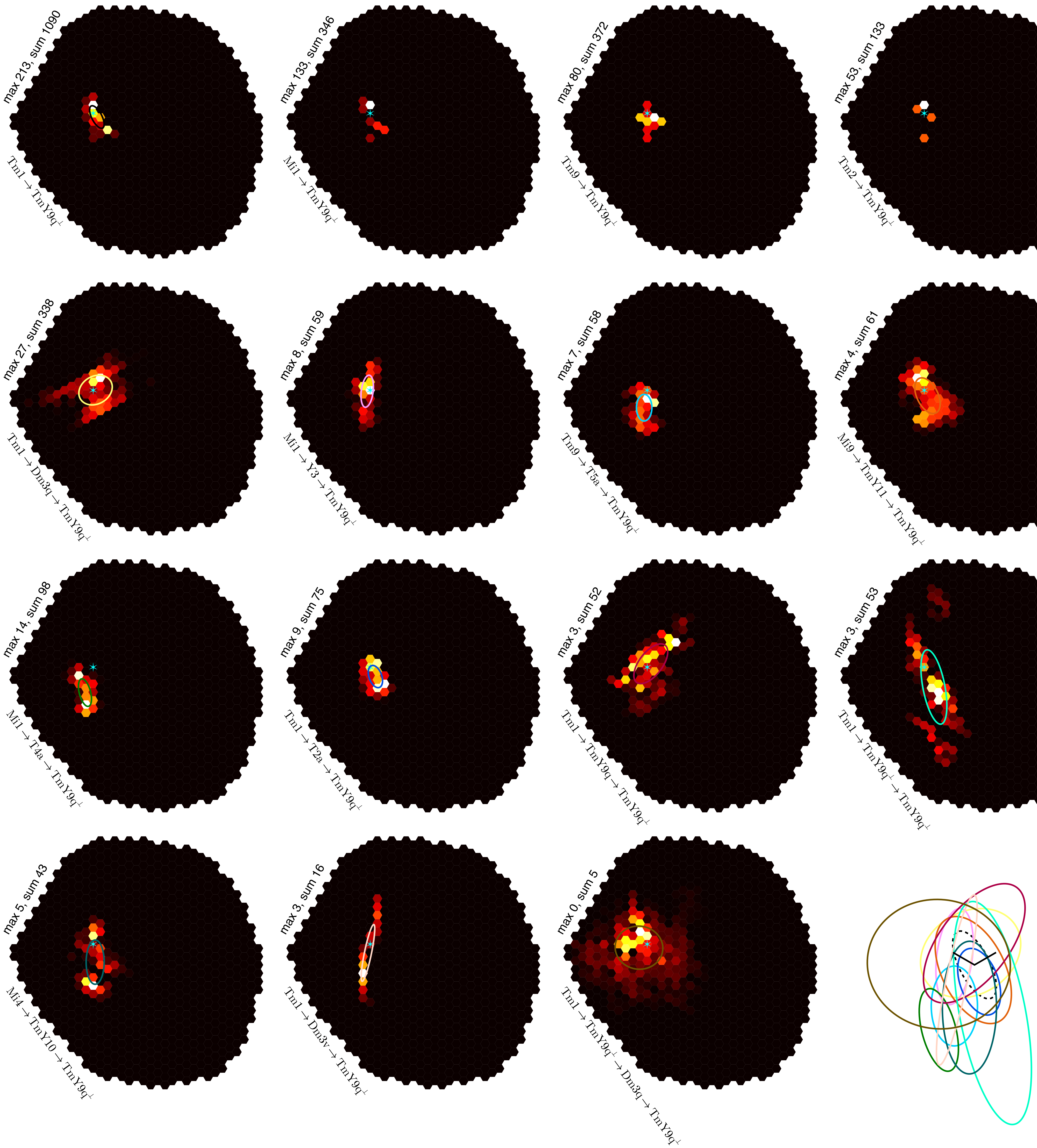
max 213, sum 1090
Tm1 → TmY9q⊥
max 133, sum 346
Mi1 → TmY9q⊥
max 80, sum 372
Tm9 → TmY9q⊥
max 53, sum 133
Tm2 → TmY9q⊥
max 27, sum 338
Tm1 → Dm3q → TmY9q⊥
max 8, sum 59
Mi1 → Y3 → TmY9q⊥
max 7, sum 58
Tm9 → T5a → TmY9q⊥
max 4, sum 61
Mi9 → TmY11 → TmY9q⊥
max 14, sum 98
Mi1 → T4a → TmY9q⊥
max 9, sum 75
Tm1 → T2a → TmY9q⊥
max 3, sum 52
Tm1 → TmY9q → TmY9q⊥
max 3, sum 53
Tm1 → TmY9q⊥ → TmY9q⊥
max 5, sum 43
Mi4 → TmY10 → TmY9q⊥
max 3, sum 16
Tm1 → Dm3v → TmY9q⊥
max 0, sum 5
Tm1 → TmY9q⊥ → Dm3q → TmY9q⊥
